# Burden, duration and costs of hospital bed closures due to acute gastroenteritis in England per winter, 2010/11–2015/16

**DOI:** 10.1016/j.jhin.2017.05.015

**Published:** 2017-09

**Authors:** F.G. Sandmann, M. Jit, J.V. Robotham, S.R. Deeny

**Affiliations:** a Department of Infectious Disease Epidemiology, London School of Hygiene and Tropical Medicine, London, UK; b Modelling and Economics Unit, Public Health England, London, UK; c The Health Foundation, London, UK

**Keywords:** Burden of disease, Length of stay, Hospital costs, Gastroenteritis, Norovirus, Outbreaks

## Abstract

**Background:**

Bed closures due to acute gastroenteritis put hospitals under pressure each winter. In England, the National Health Service (NHS) has monitored the winter situation for all acute trusts since 2010/11.

**Aim:**

To estimate the burden, duration and costs of hospital bed closures due to acute gastroenteritis in winter.

**Methods:**

A retrospective analysis of routinely collected time-series data of bed closures due to diarrhoea and vomiting was conducted for the winters 2010/11 to 2015/16. Two key issues were addressed by imputing non-randomly missing values at provider level, and filtering observations to a range of dates recorded in all six winters. The lowest and highest values imputed were taken to represent the best- and worst-case scenarios. Bed-days were costed using NHS reference costs, and potential staff absence costs were based on previous studies.

**Findings:**

In the best-to-worst case, a median of 88,000–113,000 beds were closed due to gastroenteritis each winter. Of these, 19.6–20.4% were unoccupied. On average, 80% of providers were affected, and had closed beds for a median of 15–21 days each winter. Hospital costs of closed beds were £5.7–£7.5 million, which increased to £6.9–£10.0 million when including staff absence costs due to illness.

**Conclusions:**

The median number of hospital beds closed due to acute gastroenteritis per winter was equivalent to all general and acute hospital beds in England being unavailable for a median of 0.88–1.12 days. Costs for hospitals are high but vary with closures each winter.

## Introduction

In healthcare settings, acute gastroenteritis (i.e. diarrhoea and vomiting) is a common source of disruption for routine care due to the sudden onset of symptoms, and the potential for enteric pathogens to cause outbreaks of an infectious nature [1]. In 2004, hospital outbreaks of acute gastroenteritis were estimated to cost the National Health Service (NHS) in England £115 million annually [2]. The main elements contributing to this cost were a decrease in the supply of available beds (when ‘closed’ due to acute gastroenteritis, i.e. these beds were unavailable for admissions) and staff absence due to illness. Between 2009 and 2011, Public Health England recorded a mean of 15,000 bed-days lost and 3400 cases among staff per year from voluntarily reported hospital outbreaks (primarily norovirus) [3]. This underestimates the national burden given regional variation in reporting, leading to an estimated under-ascertainment of approximately 20% [4].

Due to increased demand each winter, the impact of reduced numbers of available beds in hospitals is greater than in the rest of the year. In England, the NHS has been monitoring the performance of all acute hospital trusts each winter since 2010/11, which includes mandatory reporting of any bed closed due to diarrhoea and vomiting on weekdays [5]. These compulsory reports allow for a comprehensive overview of the impact of all causes of acute (infectious and non-infectious) gastroenteritis on bed-days lost nationwide for six winters across seven years.

This study aimed to provide updated estimates for the burden, duration and costs of all forms of acute gastroenteritis affecting the availability of hospital beds in England during winter. In addition to investigating the duration of closure, this study also explored whether the outbreak duration of infectious gastroenteritis could be traced in the data by following conventional definitions for outbreaks of norovirus [6], [7], [8], which has become the key enteric pathogen across all ages worldwide, particularly in countries that have introduced rotavirus vaccination (such as the USA) [9], [10]. This study also compared estimates across winters in order to provide insights into variation across the whole hospital system.

## Methods

### Data

Using NHS England's winter situation reports for 2010/11 to 2015/16, all available records of occupied and unoccupied beds closed due to diarrhoea and vomiting, and the total number of general and acute beds available (including escalation beds but excluding maternity and mental health beds) were obtained [5]. Records were only available at the level of trusts, which may contain multiple hospitals and/or wards within a hospital. Bed figures were reported on weekdays alone (reflecting the number for the previous day), and figures for weekends and bank holidays reflect the last day of that respective period [5]. Suspected errors in reporting or miscoding encountered in the data were treated as missing values (*N* = 0.54%; see Discussion).

### Statistical analysis

Two key issues in the data were addressed for a reliable comparison across winters. First, one-third of values were missing due to weekends and bank holidays. To account for this, values were imputed at provider-level through last observation carried forward (LOCF) and next-observation carried backward (NOCB). Therefore, records for Thursdays were carried forwards to inform the missing values for Fridays and Saturdays (with LOCF), and records for Sundays were carried backwards to impute values for Saturdays and Fridays (i.e. NOCB). To avoid biasing results systematically upwards when closed beds were recorded before but not after the missing values (and vice versa), the lowest value imputed with either LOCF or NOCB was considered as the conservative best-case scenario. Similarly, the highest value imputed with either imputation strategy was considered as the worst-case scenario.

Second, recording lengths and periods are determined flexibly by NHS officials each winter, and varied from 13 to 21 weeks between November and March. The analysis was restricted to an overlapping range of dates recorded in all six winters (30^th^ November–20^th^ February; i.e. 83 days or nearly 12 weeks) after imputing missing values.

Descriptive statistics are provided using the median and interquartile range (IQR) to highlight variation between winters. Pearson's correlation coefficient was calculated to investigate the relationship between the number of unoccupied and occupied beds closed per day. To visualize any trend across winters, locally weighted regression curves across all winters, and linear regression curves interrupted between the winters of 2012/13 and 2013/14 were fitted to the time-series of all beds closed (with highest imputations). Data (with lowest imputations) were also scaled to the highest daily number per winter using the unadjusted periods (between 13 and 21 weeks) to identify within-winter variations.

### Quantifying the duration of bed closures

The number of consecutive days of bed closures was counted per provider in the imputed datasets to analyse the duration of bed closures.

In order to explore whether outbreaks of infectious gastroenteritis could be traced in the data, conventional definitions for outbreaks of norovirus as the key enteric pathogen were followed (i.e. more than one case for more than one day; symptom onset within ±48 h) [6], [7], [8]. First, records of isolated occurrences of one bed closed for one day and no other closures within ±48 h were removed. Next, bed closures reported within ±48 h were grouped and analysed as part of one sustained outbreak. In scenario analyses, sequences with beds being closed on the first or last day of recording were removed, as well as sequences with beds being closed on the second (to last) day to account for the ±48-h period. Given that the removal of censored durations biases results when excluding the longest-lasting closures, this study also explored inclusion of the duration of outbreaks truncated to the overlapping range of dates from the raw data, where possible, and only removed the remaining censored outbreaks (i.e. for those two winters that defined the start and end date of the filtered range of dates; see Appendix A, online supplementary material).

### Cost analysis

The financial costs of acute gastroenteritis from the perspective of hospitals was estimated based on the number of unoccupied bed closures and staff absence costs.

Financial costs for hospitals arise from revenue losses of closed (unoccupied) beds as no (additional) patients can be treated in these beds. Occupied bed closures do not represent a financial loss for hospitals in the same sense; this is discussed further in the Discussion.

This study also considered the costs of staff absences given their importance, although they are not recorded in NHS England's winter situation reports. Staff absence costs as a proportion of the total costs from previous studies of outbreaks of acute gastroenteritis in England and Scotland were 0.245 and 0.174, respectively [2], [11].

The total financial and economic costs per winter were estimated to be:

(1) $${\text{TC}}_{\text{i}}={\text{BD}}_{\text{i}}\text{*C*}1\text{/}\left(1-{\text{S}}_{\text{i}}\right)$$

where TC represents total costs, BD represents the median total of all occupied and unoccupied bed closures, C represents the average NHS reference costs for elective and non-elective inpatient excess bed-days in 2014/15 [12], S represents the proportion of staff absence costs, and i represents the lowest and highest values (read: imputation and proportion) for the best-to-worst case scenarios.

All analyses were performed in R Version 3.2.2 using RStudio [13].

## Results

### Winter burden of hospital beds closed due to acute gastroenteritis

On average, 80% of general and acute NHS hospital trusts have bed closures due to diarrhoea and vomiting each winter. The median number of beds closed per winter was 88,000 (IQR 71,000–123,000) to 113,000 (IQR 88,000–151,000) with the lowest-to-highest imputation. Of these, 19.6–20.4% were unoccupied, respectively, with a median of 17,000 (IQR 13,000–23,000) to 23,000 (IQR 17,000–31,000) beds per winter. A strong positive correlation was found between occupied and unoccupied beds closed per day (best case *r* = 0.91, worst case *r* = 0.89).

During winter, the daily total general and acute hospital capacity in England was reported as a median of 100,000 (IQR 99,500–101,000) to 101,000 (IQR 99,900–102,000) bed-days with the lowest-to-highest imputation. Of these, 1.1–1.3% were closed due to diarrhoea and vomiting.

**Figure 1:**
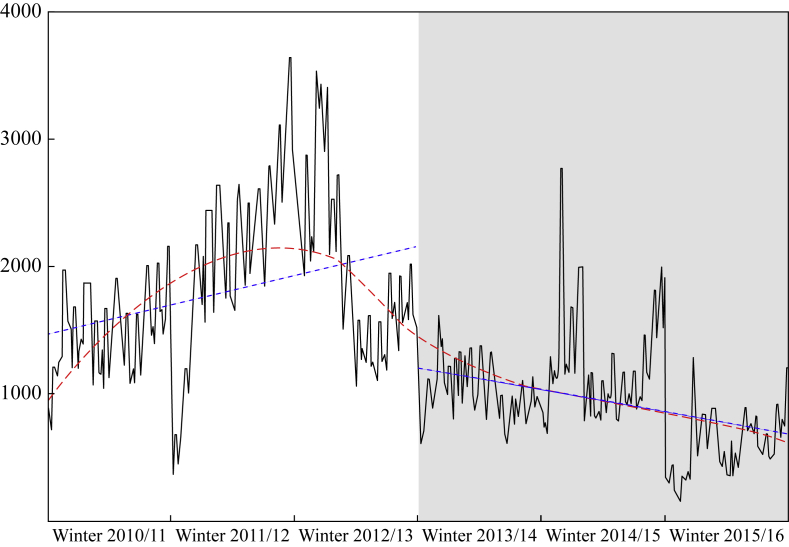
Time trend of the number of hospital beds closed due to diarrhoea and vomiting in England across winters 2010/11 to 2015/16 (30^th^ November–20^th^ February). The black line represents the data (with highest imputations), the red dashed line represents the locally weighted regression fit, and the blue dashed line represents the linear regression fit (interrupted between winters 2012/13 and 2013/14).

The highest numbers of occupied and unoccupied bed closures were found for the winters of 2011/12 and 2012/13, irrespective of the imputation scenario (see Table I). The number of beds closed has declined from a peak of 135,000–168,000 in 2011/12 to 37,800–50,100 in 2015/16, with a corresponding trend for unoccupied beds. Since 2013/14, the level of the slope of the linear fit has decreased, and its direction has become negative, even for the data with the highest imputations (see Figure 1), representing a decline in bed closures due to diarrhoea and vomiting in recent winters. Furthermore, when scaling the data to the day with the highest number of beds closed each winter (for the unadjusted periods), a peak occurred either in December or February in most winters, even when using the lowest imputations, occasionally with peaks in both months, next to an upwards linear trend for most winters (Figure A, see online supplementary material).

**Table I:** Hospital beds closed due to diarrhoea and vomiting in England, 2010/11 to 2015/16 (30^th^ November–20^th^ February)

Winter season	2010/11	2011/12	2012/13	2013/14	2014/15	2015/16
No. of trusts closing beds, per winter						
Trusts affected (% of total)	129 (78.2)	138 (84.1)	134 (84.3)	127 (80.9)	128 (83.7)	101 (66.4)

No. of beds closed, per winter						
Best case	98,500	135,000	131,000	68,600	78,100	37,800
Worst case	123,000	168,000	161,000	83,800	102,000	50,100

No. of unoccupied beds closed, per winter						
Best case	20,800	25,100	24,100	13,300	13,800	7100
Worst case	27,600	34,000	31,700	17,200	18,400	9600

No. of sustained bed closures in total, per winter						
Best case	476	512	558	503	521	388
Worst case	477	512	571	512	532	396

Duration of sustained bed closures in days, per winter						
Best case: mean (SD)	7.2 (10.0)	8.8 (12.0)	8.2 (11.9)	6.1 (8.6)	6.2 (8.4)	5.0 (7.7)
Worst case: mean (SD)	8.5 (10.1)	10.1 (12.0)	9.3 (11.8)	7.1 (8.7)	7.3 (8.3)	6.1 (7.7)
Best case: median (IQR)	3 (1–8)	4 (2–10)	4 (1–10)	3 (1–8)	3 (1–7)	2 (1–6)
Worst case: median (IQR)	5 (3–10)	6 (3–12)	5 (3–11)	4 (3–8)	5 (3–8)	4 (2–7)

No. of potential outbreaks of infectious gastroenteritis, per winter						
Best case	393	463	492	430	443	321
Worst case	348	408	437	383	419	304

Duration of potential outbreaks of infectious gastroenteritis in days, per winter						
Best case: mean (SD)	8.8 (11.5)	9.7 (12.6)	9.4 (12.7)	7.2 (9.4)	7.4 (9.2)	6.2 (9.8)
Worst case: mean (SD)	12.0 (13.9)	12.9 (14.5)	12.4 (14.0)	9.9 (11.3)	9.5 (10.7)	8.2 (10.7)
Best case: median (IQR)	4 (2–11)	5 (2–12)	4 (2–11)	4 (2–10)	4 (2–9)	3 (1–7)
Worst case: median (IQR)	7 (4–14)	8 (4–15)	6 (3–16)	6 (3–13)	6 (3–12)	5 (3–10)

Financial costs of unoccupied beds closed in million £, per winter						
Best case (excluding staff absence)	8.2 (6.8)	10.0 (8.2)	9.5 (7.9)	5.3 (4.4)	5.5 (4.5)	2.8 (2.3)
Worst case (excluding staff absence)	12.0 (9.0)	14.7 (11.1)	13.8 (10.4)	7.5 (5.6)	8.0 (6.0)	4.2 (3.2)

Economic value of occupied and unoccupied beds closed in million £, per winter						
Best case (excluding staff absence)	39.0 (32.2)	53.4 (44.1)	51.7 (42.7)	27.2 (22.5)	31.0 (25.6)	15.0 (12.4)
Worst case (excluding staff absence)	53.5 (40.4)	73.1 (55.2)	69.7 (52.6)	36.3 (27.4)	44.2 (33.4)	21.7 (16.4)

IQR, interquartile range; NHS, National Health Service; SD, standard deviation.

The total number of trusts has decreased over time from 165 in 2010/11 to 152 in 2015/16 due to mergers and restructuring in the NHS. Results do not include the scenario analyses of removing censored durations (Appendix A, see online supplementary data). For the ‘No. of potential outbreaks of infectious gastroenteritis’, the best case is higher than the worst case given that fewer sustained closures were connected. Costs represent 2014/15 values of pound sterling.

### Duration of bed closures due to acute gastroenteritis

The durations of 2960–3000 sustained closures across the six winters in the lowest-to-highest imputation were analysed. A median of three sustained closures per provider was found each winter with both imputations (full range 0–14). The median total number of days with beds closed per provider was 15 and 21 (range 0–83). Each closure lasted for a median of 3 (IQR 1–8) to 5 (IQR 3–10) days, and a mean of 7.0 (standard deviation, SD 10.1) to 8.2 days (SD 10.1).

Following the methodology to trace the potential outbreak duration of infectious gastroenteritis resulted in 2540–2300 sustained closures in the lowest-to-highest imputation. The median duration was 4 (IQR 2–10) to 6 (IQR 3–13) days and a mean of 8.3 (SD 11.1) to 10.9 (SD 12.8) days. The scenario analyses of excluding censored durations did not change the median, but the mean decreased to 8.0–10.6 or 6.7–9.1 days, depending on whether or not the duration of truncated outbreaks from the raw data were included (Appendix A, see online supplementary material).

### Costs of unoccupied beds closed due to acute gastroenteritis for hospitals

The financial cost for hospitals corresponding to the median number of unoccupied bed closures was £5.7–7.5 million in England per winter (with the range representing the lowest-to-highest imputations). This increased to £6.9–10.0 million when including staff absence due to illness (see Table I).

## Discussion

This study provides up-to-date estimates for the burden, duration and costs of all forms of acute gastroenteritis impacting hospital bed availability in England for six winters across seven years. Although only 1.1–1.3% of all general and acute beds were closed per day due to diarrhoea and vomiting in the last six winters, the total median number of beds closed per winter equalled the entire median total capacity in England per day (88,000/100,000 to 113,000/101,000 = 0.88–1.12 days). The data were subject to variation between winters. The burden of bed closures was highest in the first three winters recorded, which coincided with rotavirus vaccine introduction in July 2013 [14], [15], [16] and with the emergence of a novel norovirus strain mutation (2012/Sydney) [17], [18]. The numbers fluctuated, with the scaled data showing a second peak in mid-February in most winters (Figure A, online supplementary material).

Although the closure of occupied beds does not represent a financial revenue loss for hospitals, these beds are blocked for alternative patients and opportunity costs may arise from the lost opportunity to treat the patient who would have been admitted if the bed was available. Thus, if one assumes that the entire period that a bed was closed due to diarrhoea and vomiting is avoidable, the cost for hospitals of all bed closures (occupied and unoccupied) is £29–£37 million per winter (and £35–£49 million when including staff absence costs). However, this estimation assumes that patients who occupied closed beds would have been discharged if the beds were not closed (i.e. the length of stay of the patient would be reduced), and that there are no other clinical or operational reasons why a discharge might be delayed; considering such time-dependent biases and competing risks in the underlying discharge process was beyond the scope of this study [19].

### Comparison with previous work

The strong positive correlation between the number of unoccupied and occupied bed closures is likely to be due to closures of bays with more than one bed, which supports previous research on the impact of physical proximity of cases and the structural design of wards [8], [20].

Compared with the estimated potential outbreak duration of infectious gastroenteritis of a median of 4–6 days and a mean of 8.3–10.9 days, previous studies in England observed an average outbreak duration of 9.2 (95% confidence interval 6.5–11.9) days per ward closure to new admissions in England in 2002/03 [2], [21], and a median length of 6–7 days from Public Health England's surveillance system of voluntarily-reported norovirus outbreaks in hospitals between 2009/10 and 2014/15 [3]. Most differences may be explained by the fact that NHS England only record the winter period, instead of the whole year, and records are made per trust and not per outbreak despite the fact that several distinct outbreaks may be occurring at the same time. Nonetheless, the short incubation period and fast spread of many infectious forms of gastroenteritis may suggest local confinement [22].

In 2004, the costs of infectious gastroenteritis outbreaks in England were estimated to be £115 million per year, based on a top-down approach using data from 1994/95 and 2002/03 [2], [23]. Differences to these estimates may be explained by a calculation per annum instead of per winter, a top-down vs bottom-up approach to costing, the baseline year being a high-incidence year for norovirus with novel strain emergence, and unusually high summer activity that had a seemingly worse impact than the strain emergence in 2012 [3], [24]. Also, the number of laboratory reports for norovirus has declined in total numbers in recent years [25], possibly because of milder winters, a decline in the natural year-to-year variability of the burden overall since the late 1990s/early 2000s, or other unrelated factors like reporting practices. Also, although the actual opportunity costs may not be equivalent to the excess bed-day value, if one were to uprate the weighted average of the unit costs per bed-day for various medical specialties considered in Lopman *et al.* [2] to represent 2014/15 values [26], the estimates would increase by 12%; as such, these values can still be considered conservative.

### Strengths and limitations

To the best of the authors' knowledge, this is the first study to address both the missing values and different recording lengths in NHS England's hospital data. Previous studies have relied on raw data to estimate the number of beds closed due to diarrhoea and vomiting for four winters (2010/11–2013/14) [27], and to provide a comprehensive overview of hospital performance indicators for an overlapping range of weeks (weeks 45–6) for five winters (2010/11–2014/15) [28].

The data analysed in this study are considered, by the NHS, to be the best-available information to monitor NHS hospital performance in winter [5]. Nonetheless, the speed of collection only allows minimal validation of the raw data [5]. This may explain why a small fraction of cells in the spreadsheets were coded with a ‘-’ (*N* = 163; 0.20% of all cells), and a ‘0’ for general bed availability (N = 273; 0.34%). All of these were considered as missing values, and were thus accounted for in subsequent imputations. Despite these efforts, the estimated daily capacity of a median of 100,000–101,000 general and acute hospital beds per day is 4% lower than the official number of a median of 105,000 for general and acute beds available daily in Quarters 4 and 1 for the winters 2010/11–2015/16 [29], which may give an indication of the potential underascertainment.

Given that the data only represent a once-daily snapshot, it is possible for the status of beds to have changed within the same day (e.g. a closed occupied bed may have become a closed unoccupied bed when patients were discharged). Interestingly, when assuming that beds were not closed independently, the median number of occupied bed closures was higher for Sundays than for Thursdays (Wilcoxon signed-rank test: *P* = 0.002), while the change in occupied bed closures was not significant (*P* = 0.4). This increase in patients occupying beds due to diarrhoea and vomiting towards the end of weekends while the numbers of unoccupied beds remain constant may be a further indication for an underlying infectious cause.

Furthermore, when comparing central tendencies (mean, median) and measures of spread (SD, IQR) for the daily number of beds closed between the raw data and the imputations, the lowest imputation underestimated both central tendencies and measures of spread of the raw data, while the highest imputation overestimated them (data not shown). With the information available, it is not possible to determine where exactly the number of beds closed lies in the range of the lowest to highest imputations; this inaccuracy is unavoidable due to missing values.

Despite the declining trend in the number of beds closed per winter, the time-series may not be long enough to capture all relevant events given that cyclical norovirus strain emergence only occurs every 3–4 years [14], [15]; only the latest mutation, in 2012, was captured in the dataset. There is also background circulation of norovirus in the summer [25].

In addition, cases of acute gastroenteritis seen in hospitals may be community-acquired or of nosocomial origin [30]. Apart from in hospitals, substantial costs from infectious intestinal disease also arise in the community, and these have not been considered here [31], [32].

In conclusion, bed closures due to acute (infectious and non-infectious) gastroenteritis put general and acute NHS hospitals under pressure each winter, with all hospital beds in England being unavailable for an equivalent of 0.88–1.12 days. Approximately 19.6–20.4% of closed beds were unoccupied. If the data were collected daily over an identical time period, the imputation strategies of this analysis would have been superfluous, and thus the data would allow more precise surveillance within and across years. Future research needs to quantify the opportunity costs for patients in the NHS, and account for time-dependent biases.
