# Supplementary material for: Burden, duration and costs of hospital bed closures due to acute gastroenteritis in England per winter, 2010/11–2015/16
Source: J Hosp Infect. 2017 Sep;97(1):79–85. doi: 10.1016/j.jhin.2017.05.015 (PMC5564405; doi:10.1016/j.jhin.2017.05.015)
Supplement: Supplementary file 1 [file mmc1.docx]

**Appendix A**

*Scenario analysis for the duration of bed closures (as proxy for outbreaks of infectious gastroenteritis)*

For bed closures that started or stopped on the first or last day of recording, as well as on the second (to last) day to account for the ±48-h period, two approaches were applied. First, all these durations were removed. Second, given that more information is available on the duration of outbreaks due to truncating the raw data to an overlapping range of dates, the authors were able to include the entire duration of bed closures for most seasons (other than those defining the start and end dates, and durations extending to the start or end date of recording, which were still removed as censored).

Figure A illustrates the differences between looking at: (i) the entire duration of the recording periods, (ii) the duration of the overlapping range of dates (truncated), and (iii) the duration of the overlapping range of dates (non-truncated) with five trusts for three winters (for simplicity, the durations were kept occurring at the same days across winters), in which Winter 1 recorded the longest period (1–20 days), Winter 2 recorded day 1–15 (defining the end date for the burden analysis for a fair comparison; corresponding to 20^th^ February of winter 2010/11 in the actual data), and Winter 3 only recorded day 5–20 (defining the start date for the burden analysis for a fair comparison; corresponding to 30^th^ November of winter 2015/16 in the actual data).

When looking at the recording periods (i.e. all vertical lines in Figure A irrespective of colour and shape), durations vary due to different length and timing of recording each winter. When looking at the durations of the overlapping range of dates (i.e. all vertical lines in the grey box in Figure A), information on the actual duration is lost for Trust 1 and Trust 5. Moreover, these durations appear as censored now (as they reach the start or end date of the period), but removing them would bias results by eliminating the longest-lasting durations for which the entire length is known, at least in part (i.e. Trust 1 and Trust 5, but not Trust 4). When looking at the durations truncated at the overlapping range of dates but including those durations for which information is available (i.e. all vertical lines in the grey box plus the dashed red lines in Figure A), one can consider the information available that was otherwise ignored. Durations spanning the range of dates and for which the entire length from the recording periods is known were considered, while those outside of the filtered period were excluded (as no information is available on those across all winters). If one was to remove censored durations, the longest-lasting durations would still be lost (cf. Trust 4 in Figure A). In addition, information is incomplete for the winters that define the start or end date.


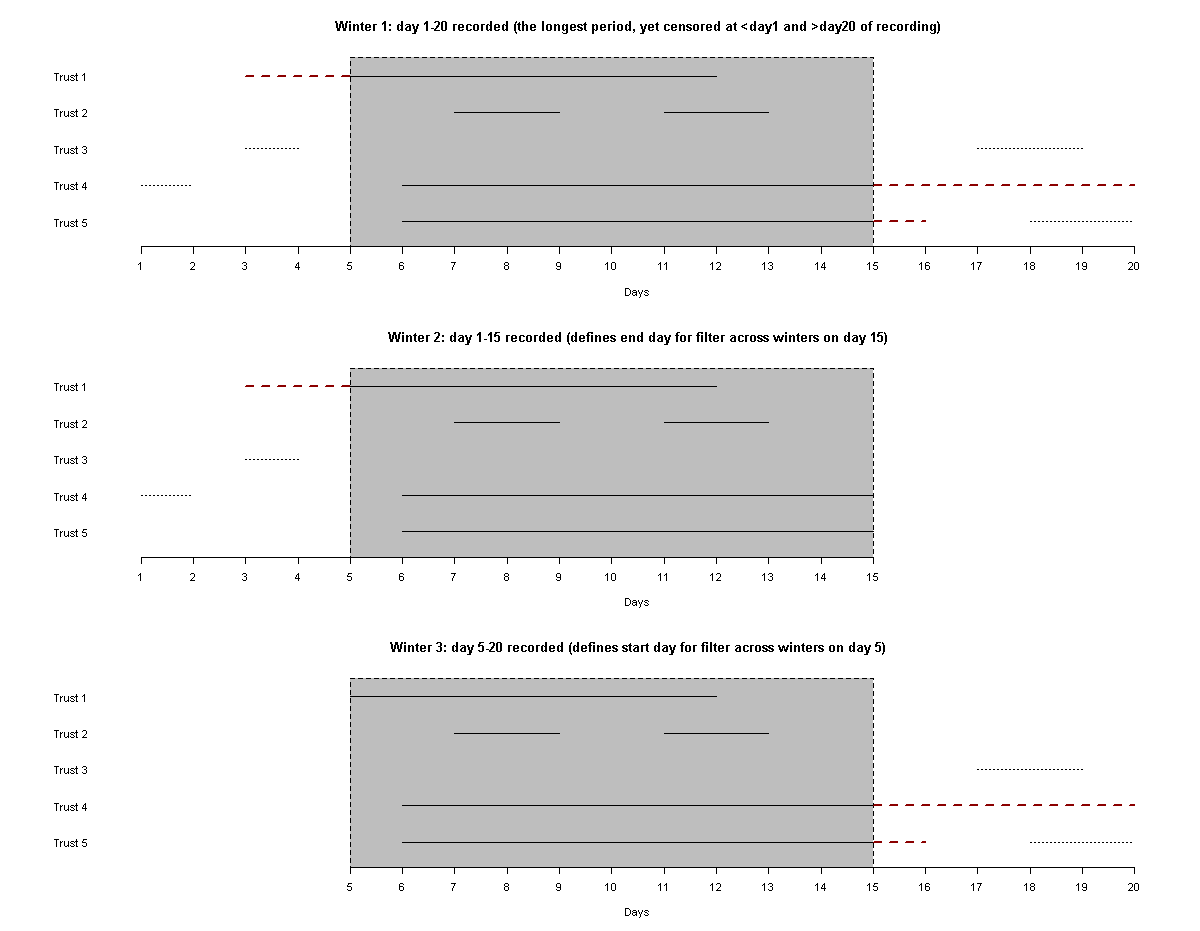
**Figure A**. Illustration of approaches to handle durations. All horizontal lines represent days with a bed closed per provider, where the black solid lines in the grey boxes represent the data for the overlapping range of dates in all three hypothetical winters, the black dashed lines represent the data outside the overlapping range of dates, and the red dashed line represents the data that could potentially be considered when looking at the overlapping range of dates.

| **Table A.** Number and length of duration of bed closures across seasons | | | | | | | | | | | | |
| --- | --- | --- | --- | --- | --- | --- | --- | --- | --- | --- | --- | --- |
|  | Recording periods | | | | Overlapping range of dates (truncated) | | | | Overlapping range of dates (non-truncated) | | | |
|  | No. of closures | Duration of closures | | | No. of closures | Duration of closures | | | No. of closures | Duration of closures | | |
| Dataset | *N* (∆) | Mean (SD) | Median (IQR) | Min, max | *N* (∆) | Mean (SD) | Median (IQR) | Min, max | *N* (∆) | Mean (SD) | Median (IQR) | Min, max |
| Raw data | 7830 | 2.77 (1.63) | 2 (1–5) | 1, 5 | 5510 | 2.75 (1.61) | 2 (1–5) | 1, 5 | 5510 | 2.75 (1.61) | 2 (1–5) | 1, 5 |
| Best case | 4240 (-3590) | 6.89 (10.7) | 3 (1–8) | 1, 147 | 2960 (-2550) | 7.02 (10.1) | 3 (1–8) | 1, 83 | 2960 (-2550) | 8.12 (12.3) | 4 (1–9) | 1, 147 |
| Worst case | 4240 (-3590) | 8.09 (10.8) | 5 (3–9) | 1, 147 | 3000 (-2510) | 8.16 (10.1) | 5 (3–10) | 1, 83 | 3000 (-2510) | 9.38 (12.2) | 5 (3–11) | 1, 147 |
| Best case: no single beds^a^ | 4060 (-180) | 7.14 (10.9) | 3 (1–8) | 1, 147 | 2850 (-110) | 7.25 (10.2) | 3 (1–8) | 1, 83 | 2850 (-110) | 8.39 (12.4) | 4 (1–10) | 1, 147 |
| Worst case: no single beds^a^ | 4170 (-70) | 8.21 (10.8) | 5 (3–10) | 1, 147 | 2960 (-40) | 8.26 (10.1) | 5 (3–10) | 1, 83 | 2960 (-40) | 9.49 (12.3) | 5 (3–11) | 1, 147 |
| Best case: connect 48 h^b^ | 3630 (-430) | 8.12 (11.9) | 4 (2–9) | 1, 147 | 2540 (-310) | 8.25 (11.1) | 4 (2–10) | 1, 83 | 2540 (-310) | 9.52 (13.5) | 4 (2–11) | 1, 147 |
| Worst case: connect 48 h^b^ | 3260 (-910) | 10.8 (13.9) | 6 (3–13) | 1, 147 | 2300 (-660) | 10.9 (12.8) | 6 (3–13) | 1, 83 | 2300 (-660) | 12.5 (15.3) | 7 (4–15) | 1, 147 |
| Best case: no censored^c^ | 3130 (-500) | 7.03 (9.0) | 4 (2–9) | 1, 75 | 2020 (-520) | 6.71 (8.2) | 4 (2–9) | 1, 61 | 2360 (-180) | 7.97 (9.9) | 4 (2–10) | 1, 75 |
| Worst case: no censored^c^ | 2740 (-520) | 9.45 (10.4) | 6 (3–12) | 1, 88 | 1710 (-590) | 9.09 (9.3) | 6 (3–12) | 1, 73 | 2100 (-200) | 10.6 (11.3) | 6 (3–13) | 1, 88 |
| SD, standard deviation; IQR, interquartile range.  ^a^Removed isolated single bed closures when no other bed closures occurred within 48 h.  ^b^Connected bed closures occurring within 48 h.  ^c^Removed censored sequences of bed closures [on first or last day of recording, or second (to last) day of recording]. | | | | | | | | | | | | |

The results for (i) the different recording periods, (ii) the overlapping range of dates (with truncated durations) and (iii) the overlapping range of dates (without truncated durations, where possible) are shown in Table A.1. As expected, the mean and median of (iii) were higher than (ii), the range of (iii) was identical to (i), and fewer censored durations were removed as a last step in (iii) than in (ii).


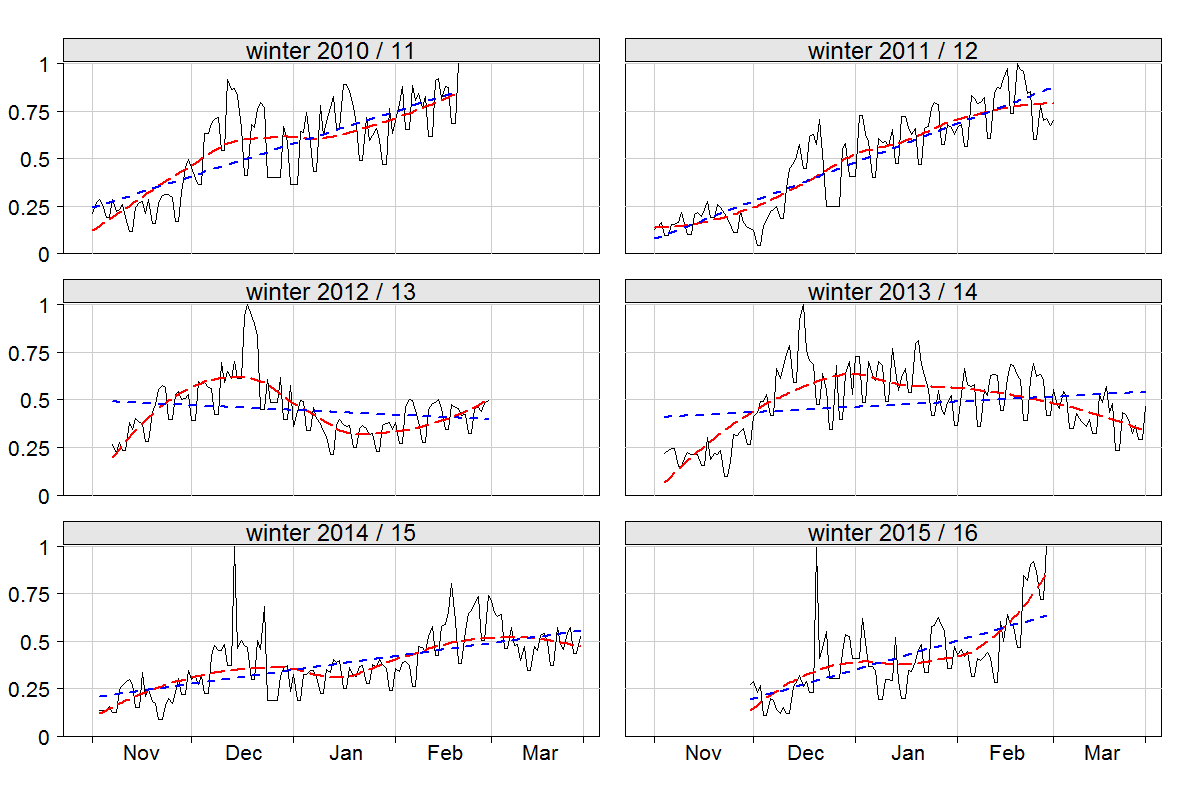
**Figure B.** Time series of the observed number (data including lowest imputations) of hospital beds closed due to diarrhoea and vomiting in England per winter, 2010/11 to 2015/16 (different recording periods). Values scaled to the highest number recorded each winter. Black line represents the data, blue dashed line represents the linear fit, and red dashed line represents the locally weighted regression fit.
